# No Increased Risk of Autoimmune Diseases Following HPV Vaccination: A Systematic Review and Meta-Analysis

**DOI:** 10.3390/vaccines13040391

**Published:** 2025-04-07

**Authors:** Filippo Alberto Ferrari, Enrico Ciminello, Marcello Ceccaroni, Matteo Pavone, Violante Di Donato, Giorgia Perniola, Pierluigi Benedetti Panici, Ludovico Muzii, Andrea Giannini, Giuseppe Vizzielli, Giorgio Bogani, Giusi Santangelo

**Affiliations:** 1 Department of Obstetrics and Gynecology, Gynecologic Oncology and Minimally Invasive Pelvic Surgery, International School of Surgical Anatomy, IRCCS “Sacro Cuore-Don Calabria” Hospital, Negrar di Valpolicella, 37024 Verona, Italy; ferrarifilippoalberto@gmail.com (F.A.F.); marcello.ceccaroni@sacrocuore.it (M.C.); 2 Italian National Institute of Health, Viale Regina Elena 299, 00161 Rome, Italy; enrico.ciminello@iss.it; 3 UOC Ginecologia Oncologica, Dipartimento di Scienze per la Salute della Donna e del Bambino e di Sanità Pubblica, Fondazione Policlinico Universitario A. Gemelli, IRCCS, 00168 Rome, Italy; matteopavone.21@gmail.com; 4 IHU Strasbourg, Institute of Image-Guided Surgery, 67000 Strasbourg, France; 5 Research Institute against Digestive Cancer (IRCAD) France, 67091 Strasbourg, France; 6 Department of Gynecological, Obstetrical and Urological Sciences, “Sapienza” University of Rome, 00184 Rome, Italy; violante.didonato@uniroma1.it (V.D.D.); giorgia.perniola@uniroma1.it (G.P.); pierluigi.benedettipanici@uniroma1.it (P.B.P.); ludovico.muzii@uniroma1.it (L.M.); andrea.giannini@uniroma1.it (A.G.); 7 Department of Medicine (DMED), University of Udine, 33100 Udine, Italy; giuseppevizzielli@yahoo.it; 8 Clinic of Obstetrics and Gynecology, “Santa Maria della Misericordia” University Hospital Azienda Sanitaria Universitaria Friuli Centrale, 33100 Udine, Italy; 9 Gynecological Oncology Unit, Fondazione IRCCS Istituto Nazionale dei Tumori di Milano, 20133 Milano, Italy; giorgiobogani@yahoo.it

**Keywords:** human papillomavirus, HPV, HPV vaccine, autoimmune disease

## Abstract

**Background:** HPV vaccination reduces the risk of anogenital warts, high-grade cervical intraepithelial neoplasia (CIN2+), and cervical cancer. To enhance immunogenicity, HPV vaccines include adjuvants such as toll-like receptor agonists, which may theoretically trigger autoimmune responses. However, existing data on this risk remain conflicting. This systematic review and meta-analysis assess the association between HPV vaccination and autoimmune disease onset in post-licensure controlled studies. **Methods:** A comprehensive literature search was conducted in Scopus, PubMed/MEDLINE, ScienceDirect, and the Cochrane Library from inception to June 2024, following PRISMA guidelines. The study protocol was registered in PROSPERO (CRD42024606834). **Results:** A total of 356 studies were identified, including cross-reference reviews. Fourteen met inclusion criteria for qualitative and quantitative analysis, encompassing 8,088,838 patients, of whom 2,041,865 received the HPV vaccine. **Conclusions:** This meta-analysis found no significant association between HPV vaccination and autoimmune disease development. However, further large-scale observational studies are needed, particularly among male recipients, as current evidence is predominantly based on female populations. Future research should also evaluate risks for specific autoimmune disorders to refine the vaccine’s safety profile.

## 1. Introduction

More than a decade after the licensure of the first human papillomavirus (HPV) vaccines, an extensive body of evidence supports the large-scale implementation of HPV immunization programs [1,2]. Clinical trials and post-marketing observational studies have consistently demonstrated the efficacy, effectiveness, and safety of available HPV vaccines: the bivalent Cervarix (targeting HPV types 16 and 18), the quadrivalent Gardasil (targeting HPV types 6, 11, 16, and 18), and the nonavalent Gardasil 9 (targeting HPV types 6, 11, 16, 18, 31, 33, 45, 52, and 58) [3,4,5,6,7,8].

HPV vaccination has significantly reduced the incidence of anogenital warts, high-grade cervical intraepithelial neoplasia (CIN2+), and cervical cancer [9,10,11,12,13,14]. These vaccines represent a major advancement in public health and cancer prevention [2]. However, despite efforts to expand immunization programs, suboptimal coverage remains a challenge. Vaccine hesitancy—fueled by misinformation, safety concerns, and public debates—continues to hinder higher immunization rates [2,15,16,17,18].

To enhance immune responses, HPV vaccines incorporate adjuvants, such as toll-like receptor agonists, which theoretically could trigger autoimmune reactions [19]. Several post-marketing studies have investigated the potential association between HPV vaccination and autoimmune diseases (ADs), yet findings remain inconsistent [20,21,22]. Many studies lack statistical power to detect rare adverse events or do not include appropriately matched control groups, limiting the reliability of their conclusions.

This systematic review and meta-analysis aimed to evaluate the association between HPV vaccination and the onset of autoimmune diseases in post-licensure studies that included a control group.

## 2. Materials and Methods

### 2.1. Protocol and Registration

This review followed the Preferred Reporting Items for Systematic Reviews and Meta-Analyses (PRISMA) guidelines and was registered in the International Prospective Register of Systematic Reviews (PROSPERO, ID CRD42024606834).

### 2.2. Search Strategy and Selection Criteria

A certified professional librarian from Biblioteca Meneghetti, University of Verona, conducted a literature search, from inception to June 2024, in the electronic databases Scopus, PubMed/MEDLINE, ScienceDirect, and the Cochrane Library. The search strategy included combinations of medical terms such as “vaccine”, “anti-papillomavirus”, “autoimmune”, “disease”, and “disorder”, in combination with “AND” and “OR” functions. The references of all identified studies were systematically reviewed to identify other eligible publications. The complete search strategy is available in Appendix A.

### 2.3. Inclusion and Exclusion Criteria

We included all full-text manuscripts published in English that met the pre-specified PICOS criteria: (P) patients vaccinated with any HPV vaccine; (I) vaccinated HPV-vaccine (C) compared to placebo or administration of other vaccines; (O) studies measuring the incidence of autoimmune adverse events related to vaccine administration; (S) randomized controlled trials and observational studies with a control arm. Studies published in English up to 30 June 2024, were considered eligible for inclusion in the present analysis. Only full-text studies providing sufficient data on study design were included. Case reports and cohort studies without a control arm were excluded.

### 2.4. Study Selection and Data Extraction

An initial screening of titles and abstracts was conducted prior to full-text evaluation. The screening process was independently conducted by two review authors (SG and FAF) and any disagreements over the eligibility of studies were resolved through discussion with a third author (EC).

A standardized form was developed and used to extract data from the studies including first author’s last name, year of publication, study design, total number of participants, age range, type of vaccines, gender distribution, number of vaccinated participants in each study arm, total autoimmune events, events in group. One review author (FFA) extracted the data from the included studies, and a second author (SG) verified the extracted data. Disagreements were resolved through discussion between the two review authors; if no agreement could be reached, a third author (MC) made the final decision.

### 2.5. Risk of Bias Assessment

Two independent reviewers (FAF and GS) evaluated the risk of bias in the included studies. For randomized controlled trials (RCTs), the updated version of the Cochrane Collaboration’s risk of bias tool (RoB 2.0) was employed, while the Risk of Bias in Non-Randomized Studies of Interventions (ROBINS-I) tool was applied to non-randomized studies [23,24]. The overall quality of the evidence was appraised using the Grading of Recommendations Assessment, Development, and Evaluation (GRADE) framework, in its latest version [25].

### 2.6. Statistical Analysis

To assess the safety of HPV vaccines, we extracted data on the number of subjects experiencing an autoimmune disease (AD) in the vaccine group and the related placebo group from each study included in the analysis. We considered experiencing ADs overall as the main analysis and performed sub-analyses with specific focus by category of disease according to the following list: gastrointestinal ADs; thyroid ADs; musculoskeletal ADs; neurological ADs; dermatologic ADs; diabetes mellitus type I. Only studies with information on a specific sub-category were included in the related sub-analysis. For each category, we calculated risk ratios (RR) and their 95% confidence intervals (CI_95%_). The effect of vaccine on adverse effects was estimated by statistically combining information from different studies via a random effects model. The results of the model and the information extracted from each study for each category were reported by forest plot. Heterogeneity between the included studies was assessed using I2 statistics and the Cochran Q test. I2 values were interpreted as low (25–50%), moderate (50–75%), and high (75% and higher) levels of heterogeneity. A meta-regression was conducted and a leave-one-out validation step was implemented to measure the impact of study design or cohort-baseline features and to check for studies affecting overall heterogeneity. Uni- and multivariate adjustments were performed with study design characteristics, namely treatment (Cervarix, Gardasil, either), control group (unexposed, other vaccines, placebo), and months of follow-up, and with baseline characteristics, namely geographical area (EU, US, world), sex and age (adults included). The threshold for statistical significance was fixed equal to 0.05. All statistical analyses were performed by the software R version 4.4.2 (31 October 2024 ucrt)—“Pile of Leaves”.

## 3. Results

### 3.1. Study Selection

**Figure 1:**
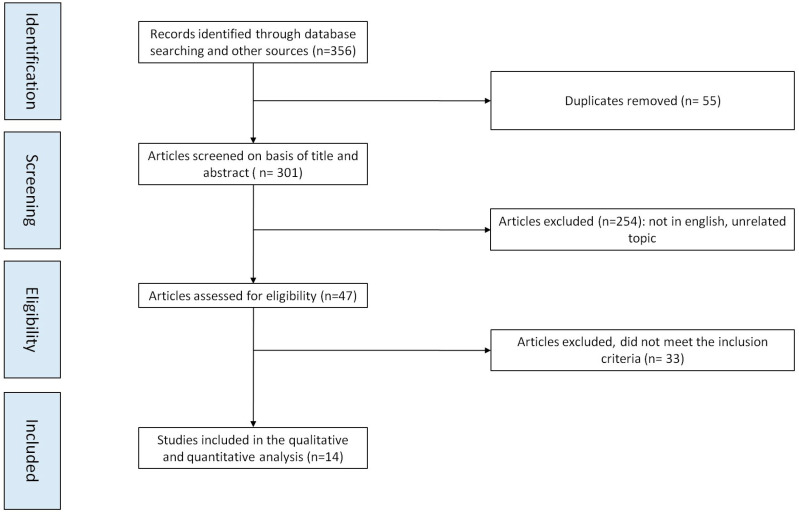
Flow chart of study selection.

A flowchart of the literature search is shown in Figure 1. Our literature search identified 356 papers, including studies identified with cross-reference review. Duplicates were excluded, and after the title and abstract screening, 47 potentially relevant articles were identified and underwent full-text assessment for eligibility. A total of 33 studies were excluded because they failed to meet the inclusion criteria. The remaining 14 studies were included in the qualitative and quantitative analysis, comprising a total of 8,088,838 patients of which 2,041,865 were HPV vaccinated [26,27,28,29,30,31,32,33,34,35,36,37,38,39].

The characteristics of the studies are summarized in Table S1. Among the 14 included studies, the bivalent Cervarix vaccine was used in one study [26], the quadrivalent Gardasil vaccine was used in nine studies [27,28,29,32,33,34,35,36,38], and both types of vaccines were used in the remaining studies [30,31,37,39]. All the included studies included a control group. Specifically, four studies involved subjects administered with other vaccines [16,19,23,29], nine studies involved unexposed subjects [27,28,30,31,34,35,36,37,38], and one study used a placebo administration [32]. Most studies included female subjects, one study included only male subjects [38], and five studies included patients of both sexes [26,27,29,33,39]. The age range of the included subjects is broad, varying depending on the study from 9 to 45 years [33] with five studies enrolling patients only under 18 years old [26,36,37,38,39]. To better interpret the results, we analyzed the data by focusing on the following main areas, as proposed by previous studies: gastrointestinal, thyroid disease, musculoskeletal, neurological, dermatological, and other diseases. Within these categories, we conducted sub-analyses for individual autoimmune diseases whenever possible.

### 3.2. Risk of Bias Assessment

**Figure 2:**
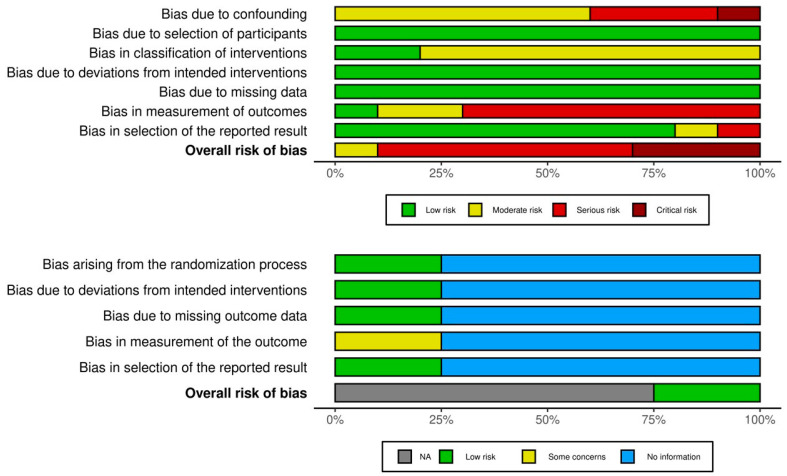
Summary of risk of bias assessment.

We summarized the findings of our risk of bias assessment in Figure 2 and Supplementary Table S3. The risk of bias in the RCT by Bi et al., was considered to be low for most indicators, but there were some concerns regarding the selection of the reported results. We were not able to assess the risk of bias in the pooled analyses [31,32,33], because of the complexity of the pooled design and lack of reporting of the key indicators for the risk of bias assessment. The risk of bias in 6 of 10 non-randomized studies was assessed as being serious; three studies were assessed as being at critical risk and one study was assessed at moderate risk of bias. The risk of bias was mostly introduced by the critical risk of confounding, or because of the outcome measurement. Risk of bias because of the classification of the intervention (vaccination status) was moderate when based on a registry and critical when self-reported.

### 3.3. Overall AD Risk

**Figure 3:**
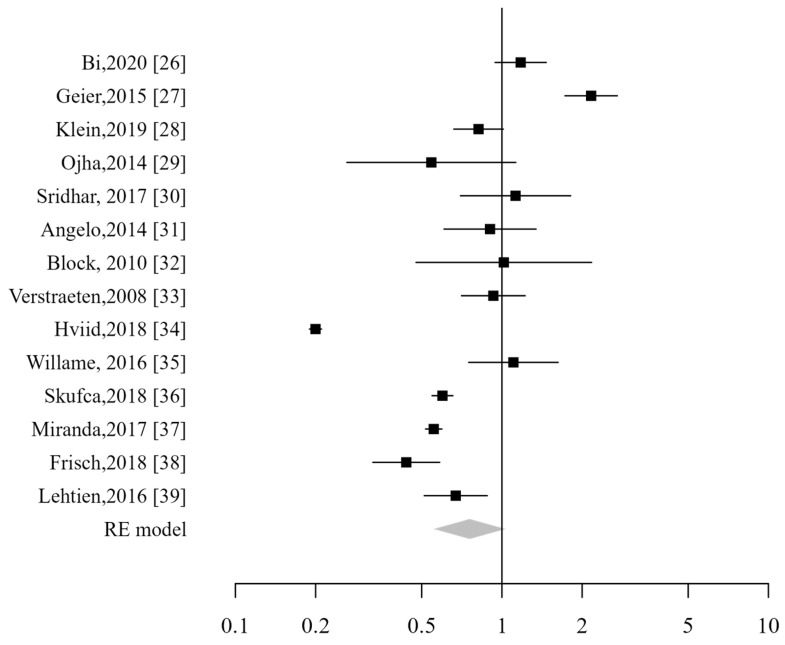
Forest plot of overall ADs [26,27,28,29,30,31,32,33,34,35,36,37,38,39].

All 14 studies were included in the pooled analysis, which did not demonstrate an increased risk for patients vaccinated against HPV, with a combined relative risk of 0.76 (CI_95%_: 0.55–1.03). The analysis demonstrated a high heterogeneity (I2 99%; *p* < 0.001; Figure 3). Excluding studies that included male subjects did not alter the analysis results. The sensitivity analysis showed that the pooled risk estimates remained stable even when any individual study was excluded.

### 3.4. Gastrointestinal AD Risk

**Figure 4:**
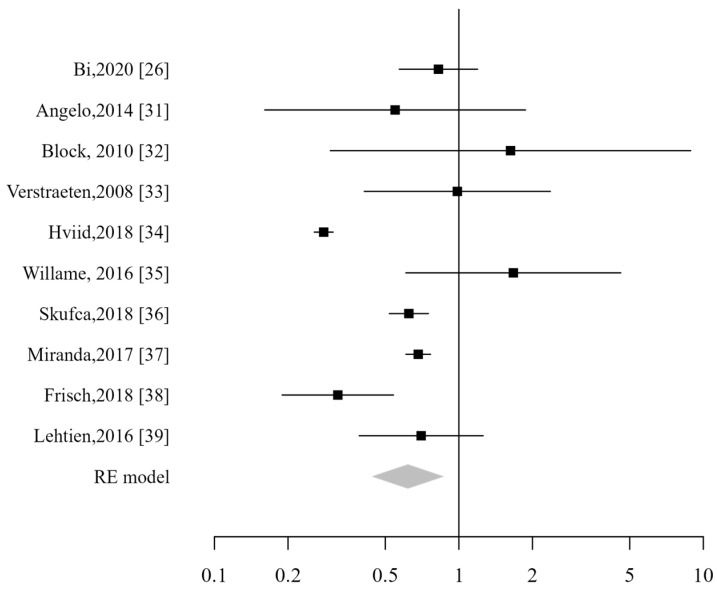
Forest plot of gastrointestinal ADs [26,31,32,33,34,35,36,37,38,39].

Ten studies were included in the analysis, and no significantly increased risk related to the HPV vaccine was demonstrated (RR 0.62; CI_95%_: 0.44–0.87) (Figure 4). In the specific sub-analysis for each pathology, no increased risk was found for Crohn’s disease (RR 0.46; CI_95%_: 0.32–0.66), celiac disease (RR 0.48; CI_95%_: 0.35–0.65), or ulcerative colitis (RR 0.61; CI_95%_: 0.37–1.01). Similarly, no increased risk was observed for proctitis, although only two studies reported data on this condition.

### 3.5. Thyroid AD Risk

**Figure 5:**
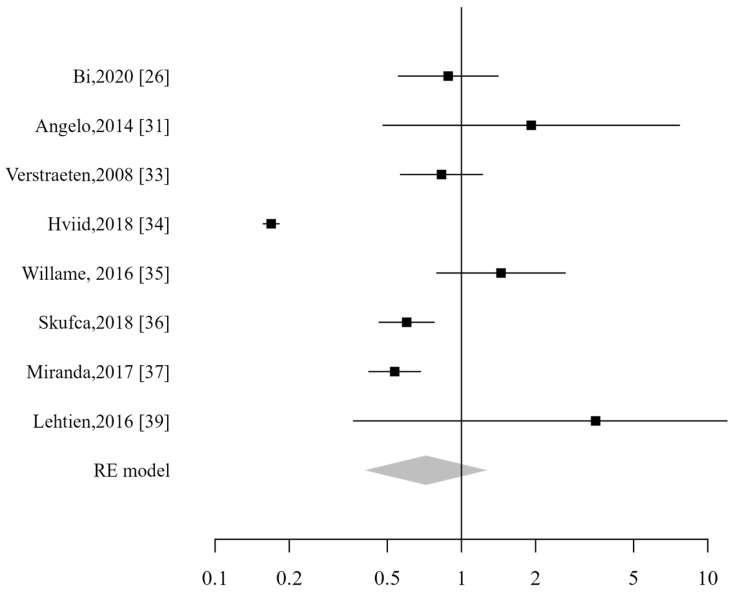
Forest plot of thyroid ADs [26,31,33,34,35,36,37,39].

Considering all events reported in the included studies, we conducted an analysis of the overall risk of developing thyroid disease in the patient cohorts. This macro group comprised both common autoimmune thyroid conditions (Hashimoto’s thyroiditis and Graves’ disease) and cases of hypothyroidism and hyperthyroidism reported during the follow-up periods of the individual studies. Our analysis did not demonstrate an increased risk in vaccinated individuals (RR 0.72; CI_95%_: 0.4–1.28) (Figure 5). To further elucidate these findings, we performed a sub-analysis specifically focused on autoimmune thyroiditis. This sub-analysis also revealed no increased risk of developing these adverse events in the vaccinated population (RR 0.96; CI_95%_: 0.44–2.12). These results suggest that HPV vaccination does not contribute to an elevated risk of thyroid-related conditions.

### 3.6. Musculoskeletal or Systemic AD Risk

**Figure 6:**
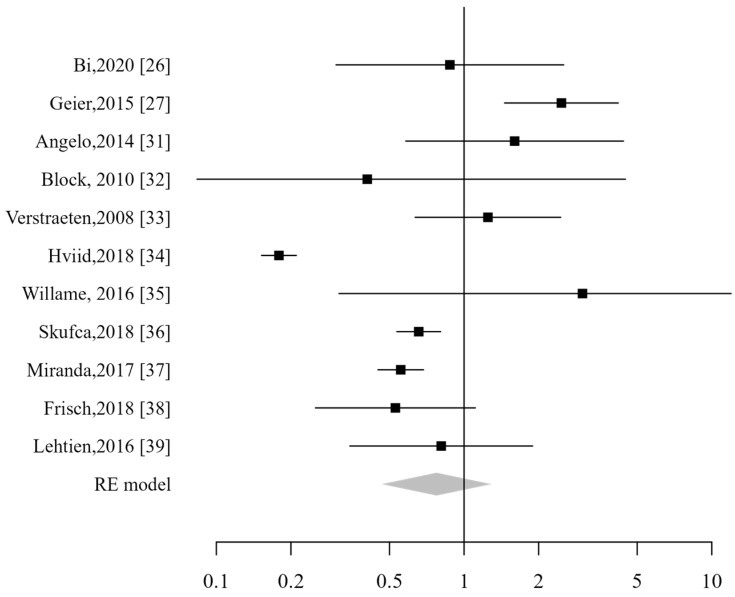
Forest plot of musculoskeletal ADs [26,27,31,32,33,34,35,36,37,38,39].

Eleven studies reported the risk of musculoskeletal disorders, and the pooled analysis did not demonstrate an increased risk for patients who received the HPV vaccination (RR 0.77; CI_95%_: 0.47–1.29) (Figure 6). When evaluating the risk associated with specific conditions within this category, the analysis did not show an increased risk related to HPV vaccination for rheumatoid arthritis (RR 0.61; CI_95%_: 0.31–1.23) and autoimmune arthritis (RR 1.01; CI_95%_: 0.5–2.03). While two studies provided data on psoriatic arthritis, the analysis for this specific condition was not feasible due to the limited data available. Consequently, no definitive conclusions can be drawn regarding the risk of psoriatic arthritis following HPV vaccination.

### 3.7. Neurological AD Risk

**Figure 7:**
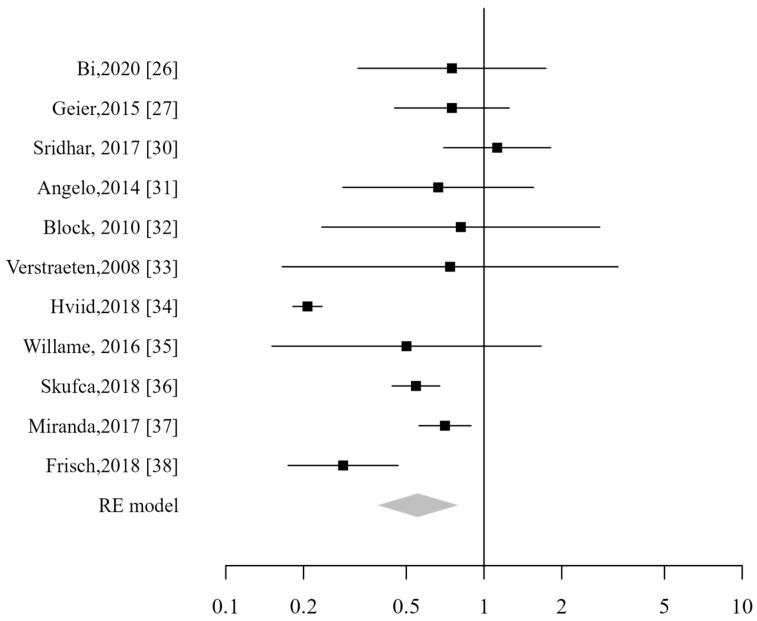
Forest plot of neurological ADs [26,27,30,31,32,33,34,35,36,37,38].

Eleven studies investigated the risk of neurological autoimmune diseases in relation to HPV vaccination, and the pooled relative risk for neurological ADs was 0.55 (CI_95%_: 0.39–0.8). (Figure 7). Ojha et al. specifically examined the risk of Guillain–Barré syndrome associated with HPV vaccination [29], while Sridhar focused on the risk of neuroinflammatory disorders [30]. Further analyses, considering the specific types of neurological ADs, indicated that HPV vaccination was not linked to an increased risk of neuroinflammatory disorders (RR 0.88; CI_95%_: 0.64–1.2)., Guillain–Barré syndrome (RR 0.92; CI_95%_: 0.54–1.58) or multiple sclerosis (RR 1.38; CI_95%_: 0.3–6.42).

### 3.8. Dermatologic ADs Risk

**Figure 8:**
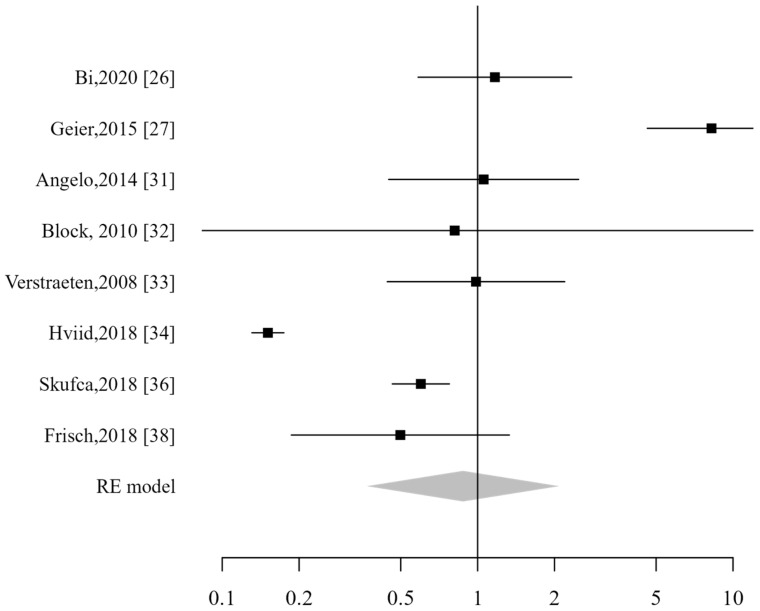
Forest plot of dermatologic ADs [26,27,31,32,33,34,36,38].

Seven studies examined the risk of dermatological ADs in relation to HPV vaccination, with a combined relative risk 0.99 (CI_95%_: 0.31–3.2) (Figure 8). When considering specific types of dermatological ADs, there was no significant increase in risk for Raynaud syndrome (RR 0.57; CI_95%_: 0.21–1.57), psoriasis (RR 0.38; CI_95%_: 0.09–1.58) and vitiligo (RR 0.56; CI_95%_: 0.12–2.61). Geier et al. reported an increased risk of alopecia in individuals vaccinated against HPV [27]. However, since only two studies assessed the risk of developing alopecia, it was not possible to perform a pooled analysis to estimate an overall risk.

### 3.9. Other ADs Risk

**Figure 9:**
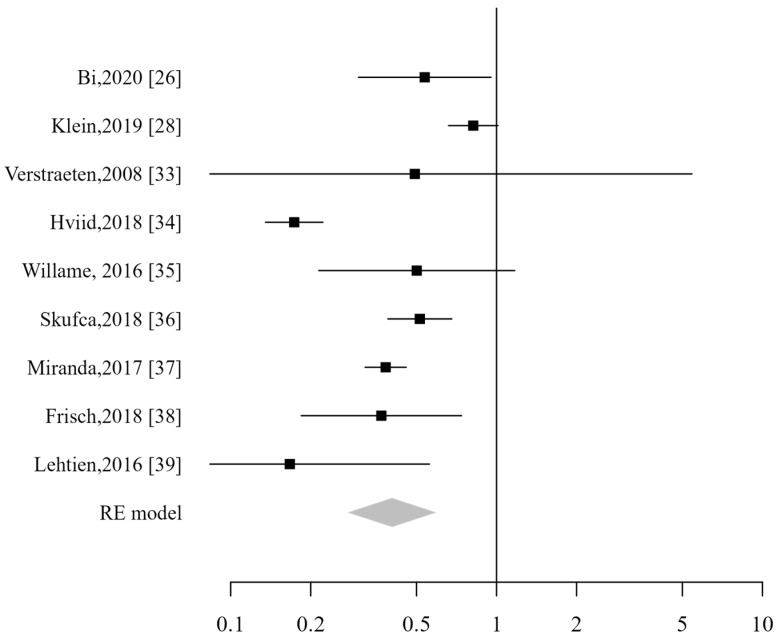
Forest plot of diabetes mellitus type ADs [26,28,33,34,35,36,37,38,39].

A pooled analysis was conducted to assess the risk of specific autoimmune diseases following HPV vaccination. The risk of systemic lupus erythematosus (SLE) was evaluated based on six studies, showing no significant association (RR 0.9; CI_95%_: 0.27–2.99). For type 1 diabetes mellitus (T1DM), nine studies were included in the analysis, indicating no increased risk (RR 0.4; CI_95%_: 0.28–0.59) (Figure 9). Additionally, the overall risk of vasculitis was assessed across nine studies, with results confirming no significant association (RR 0.83; CI_95%_: 0.44–1.57).

### 3.10. Ancillary Analyses

Tables S1 and S2 show the results of meta-regression for overall adverse events including baseline and study design characteristics, respectively. Adjusting for baseline characteristics only partially affects heterogeneity, which keeps a high level with I2 statistic always higher than 95% without a statistically significant effect of explored features. The same is obtained when adjusting for study design features, with I2 statistic always higher than 97%. Table S3 shows the results of the leave-one-out analysis. Repeating the overall meta-analysis while leaving out iteratively one study for validation showed that none of the studies included in the analysis had a major impact on heterogeneity and on pooled effect size. Excluding Hviid [34] from the analysis leads to a decrease in heterogeneity, which, however, remains significantly high (*p* < 0.001), with I2 statistics reaching 94%. Tables S1–S3 are available in Supplementary Materials: Supplementary_Materials_Ancillary_Analises.xlsx.

## 4. Discussion

The primary objective of this meta-analysis was to assess the incidence of autoimmune conditions related to HPV vaccination, including only studies with control groups. A total of 14 studies were included in the qualitative and quantitative analysis, comprising 8,088,838 patients, of whom 2,041,865 had received the HPV vaccine. No increased overall risk of developing autoimmune diseases was identified in vaccinated individuals, regardless of whether they received the bivalent or quadrivalent HPV vaccine. Adjustments and validation steps confirm the robustness of such results. These findings align with previous reports in the literature, further supporting the safety profile of HPV vaccination [20,21,40].

Historically, concerns about vaccine safety have led to vaccine hesitancy, which has contributed to the resurgence of preventable diseases [9,41]. Consequently, studies reassuring about vaccine safety are crucial for public health. Reviews and meta-analyses typically have found no associations between vaccines and adverse events like autism and leukemia [42]. While no significant association between HPV vaccination and autoimmune diseases has been identified, rare associations between other vaccines and adverse events have been observed, such as febrile seizures following MMR or MMRV vaccination and intussusception after rotavirus vaccination [43].

Our findings did not demonstrate any increased risk of gastrointestinal autoimmune diseases. In our study, 14 studies were included in the qualitative and quantitative analysis, comprising a total of 8,088,838 patients of which 2,041,865 were HPV vaccinated. These results are consistent with the findings of previous metanalysis [21,22], but in disagreement with those of Jiang et al. [20]. However, Jiang et al. reported a trend towards an increased risk of gastrointestinal autoimmune diseases, which did not reach statistical significance (OR = 1.06; CI_95%_: 0.99–1.14). Despite our analysis indicating a protective effect for Crohn’s disease (RR = 0.42; CI_95%_: 0.3–0.72), the actual clinical impact should be interpreted with caution. Only two studies have assessed the risk of autoimmune proctitis, finding an increased risk; however, it was not possible to perform a pooled analysis [26,33].

We did not find an increased risk of developing thyroid diseases or autoimmune thyroiditis. The meta-analysis by Jiang et al. indicated an increased risk for Hashimoto’s thyroiditis (OR = 1.22, CI_95%_: 1.09–1.36) but the authors themselves considered that part of this risk might be due to the inclusion of two studies that enrolled only adult patients, suggesting that the symptoms of Hashimoto’s thyroiditis might be more evident in this age group and therefore potentially more frequently reported [20]. The same hypothesis was proposed by Willame et al., whose results on autoimmune thyroiditis were mainly driven by Chung et al. [35]. Upon further examination, the authors concluded that the majority of new onset cases were likely pre-existing and found no consistent evidence indicating a safety signal for autoimmune thyroid conditions among vaccinated individuals.

In the analysis of the musculoskeletal group, we did not find any increased risk consistent with the results of previous analyses. Among the included studies, only Geier et al. reported an increased risk of developing autoimmune diseases [27].

Hviid et al. reported an increased risk of Addison’s disease with an OR of 1.71 (CI_95%_: 0.94–3.12) [34]. However, only Verstraeten et al. provided specific data for this condition, making it impossible to assess the overall risk in a combined analysis [33].

A crucial consideration is the administration of HPV vaccination to individuals with autoimmune diseases. In the past decade, treatments with immunomodulatory and anti-tumor necrosis factor medications have been successful in achieving and sustaining remission in these patients [44]. However, these treatments also render patients more vulnerable to infections, many of which are preventable through vaccination [44,45]. Previous studies indicated a higher prevalence of latent HPV infection and cervical dysplasia in individuals with autoimmune diseases compared to healthy controls, highlighting the necessity of HPV vaccination in this group [22,46]. Despite this, there are theoretical concerns that HPV vaccination could potentially trigger disease flares or other adverse events in these patients [44]. The available data and our analysis were underpowered to investigate this aspect. However, several authors report that the HPV vaccine is well tolerated in patients with stable autoimmune conditions, without causing an increase in symptoms or disease flare-ups [19]. Data on the presence of pre-existing autoimmune conditions are not frequently reported, and the results of previous pooled analyses should be interpreted with caution due to the quality of the data and the small sample sizes.

Consequently, the strength of evidence from this meta-analysis is considered moderate according to the Grading of Recommendations Assessment, Development, and Evaluation (GRADE) criteria. However, the heterogeneity and retrospective design of some included studies highlight the need for further research with robust randomization techniques to ensure balanced baseline characteristics. Additionally, it is essential for future studies to incorporate adequate follow-up to strengthen the evidence. The primary strength of this meta-analysis is its large sample size and comprehensive search strategy, allowing for multiple analyses and enhancing the robustness of the results. This study extends the body of evidence by evaluating a broader range of ADs. The findings of this meta-analysis must be interpreted with caution due to several significant limitations. First, the number of clinical trials included that met the eligibility criteria was limited, potentially undermining the robustness and generalizability of our conclusions. Additionally, the studies were conducted across diverse geographical regions, contributing to heterogeneity in subgroup analyses, which may compromise result reliability. This variability underscores the need for further investigation into potential confounding factors, such as sex-based differences in vaccine response, age distribution, inconsistencies in reporting methodologies, and variations in the types of autoimmune diseases assessed. Furthermore, the inclusion of male patients was notably limited, making any conclusions drawn for this subgroup particularly tentative. Second, subgroup analyses for specific autoimmune diseases may be less reliable, due to the limited number of studies available for certain conditions and the rarity of individual outcomes, reducing the statistical power and robustness of the findings. More well-designed studies with larger sample sizes are necessary to thoroughly examine the association between HPV vaccination and the risk of individual autoimmune disorders. Third, only one study reported estimates for male patients reporting any association between HPV vaccination and subsequent AD risk, whereas our findings are primarily based on data from female recipients. Therefore, it is essential to confirm that the safety profile extends beyond female subjects. Finally, all studies included in this meta-analysis were conducted in Europe and North America, with no representation from Asian or African countries. Consequently, the findings cannot be generalized to populations in these regions. Additional observational studies from Asian and African countries are needed to provide epidemiological evidence on the influence of HPV vaccination on the risk of ADs in these populations.

## 5. Conclusions

In conclusion, the results of this meta-analysis did not demonstrate a significant association between HPV vaccination and the development of autoimmune disorders. There is a clear need for additional, larger observational studies to assess the association of HPV vaccination with ADs, particularly among male recipients, as the current evidence is predominantly based on female subjects. Moreover, further studies are essential to investigate the implications of HPV vaccines for the most commonly reported individual ADs, providing a more comprehensive understanding of the vaccine’s safety profile. Efforts to overcome barriers to HPV vaccination access and uptake are crucial to achieve the full public health benefits of HPV vaccination. By strengthening the vaccination infrastructure, enhancing education and communication strategies, we can potentially maximize the impact of HPV vaccination in reducing the burden of HPV-associated diseases.

## Data Availability

The data sets generated for this study are available on request from the corresponding author.

## Supplementary Materials

The following supporting information can be downloaded at: https://www.mdpi.com/article/10.3390/vaccines13040391/s1, Table S1. Results of adjusting for baseline characteristics; Table S2. Results of adjusting for study design characteristics; Table S3. Results of leave-one-out iterative scheme. (Refs. [26,27,28,29,30,31,32,33,34,35,36,37,38,39]).

## Appendix A

Supplementary Data. Scopus: (TITLE-ABS-KEY (vaccine OR “anti-papillomavirus”) AND TITLE-ABS-KEY (autoimmune OR disease OR disorder)). Pubmed/MEDLINE: (“vaccine” [Title/Abstract] OR “anti-papillomavirus” [Title/Abstract]) AND (“autoimmune” [Title/Abstract] OR “disease” [Title/Abstract] OR “disorder” [Title/Abstract]). ScienceDirect: (vaccine OR “anti-papillomavirus”) AND (autoimmune OR disease OR disorder). Cochrane Library: (vaccine OR “anti-papillomavirus”) AND (autoimmune OR disease OR disorder).
